# A diagnosis based on the electrocardiogram before laboratory results were available

**DOI:** 10.1007/s12471-015-0713-6

**Published:** 2015-05-13

**Authors:** S.L. Gerritse, E.H.C.C. Janssen

**Affiliations:** 1 Amphia Hospital, Molengracht 21, 4818 CK Breda, The Netherlands; 2 Erasmus MC, Rotterdam, The Netherlands

The ECG showed a regular rhythm with a widened QRS complex, in a sine-wave pattern. There was a fusion of QRS-T and absence of P waves, suspicious for severe hyperkalaemia. His serum potassium level was 10.0 mmol/l (confirmed with an arterial blood gas analysis) with a metabolic acidosis (pH 7.09, bicarbonate 11 mmol/l, pCO_2_ 4.8 kPa) and acute kidney failure (creatinine level of 363 µmol/l). The hyperkalaemia was treated with calcium gluconate, insulin and glucose intravenously. We admitted the patient to the intensive care unit to start continuous venovenous haemofiltration. With normalisation of his potassium level, the ECG proved to be normal. His hyperkalaemia was thought to be secondary to (pre-renal) kidney failure by dehydration and the use of perindopril.

